# DC Series Arc Fault Detection in Electric Vehicle Charging Systems Using a Temporal Convolution and Sparse Transformer Network

**DOI:** 10.3390/s26061897

**Published:** 2026-03-17

**Authors:** Kai Yang, Shun Zhang, Rongyuan Lin, Ran Tu, Xuejin Zhou, Rencheng Zhang

**Affiliations:** 1 Key Laboratory of Process Monitoring and System Optimization for Mechanical and Electrical Equipment (Fujian Provincial Department of Education), College of Mechanical Engineering and Automation, Huaqiao University, Xiamen 361021, China; shunzhang667@stu.hqu.edu.cn (S.Z.); linrongyuan0314@stu.hqu.edu.cn (R.L.); turan@hqu.edu.cn (R.T.); zhouxj@ustc.edu (X.Z.); phzzrc@hqu.edu.cn (R.Z.); 2 Fujian Key Laboratory of Green Intelligent Drive and Transmission for Mobile Machinery, Huaqiao University, Xiamen 361021, China

**Keywords:** electric vehicle charging system, DC series arc fault, deep neural network, edge computing deployment

## Abstract

In electric vehicle (EV) charging systems, DC series arc faults, due to their high concealment and severe hazard, have become one of the important causes of electric vehicle fire accidents. An improved hybrid arc fault model of a charging system was established in Simulink for preliminary study. The results show that the high-frequency noise generated by arc faults affects the output voltage quality of the charger, and this noise is conducted to the battery voltage. Arc faults in a real electric vehicle charging experimental platform were further investigated, where it was found that, during arc fault events, the charging system provides no alarm indication, and the current signals exhibit significant large-amplitude random disturbances and nonlinear fluctuations. Moreover, under normal conditions during vehicle charging startup and the pre-charge stage, the current waveforms also present high-pulse spike characteristics similar to arc faults. Finally, a carefully designed deep neural network-based arc fault detection algorithm, Arc_TCNsformer, is proposed. The current signal samples are directly input into the network model without manual feature selection or extraction, enabling end-to-end fault recognition. By integrating a temporal convolutional network for multi-scale local feature extraction with a sparse Transformer for contextual information aggregation, the proposed method achieves strong robustness under complex charging noise environments. Experimental results demonstrate that the algorithm not only provides high detection accuracy but also maintains reliable real-time performance when deployed on embedded edge computing platforms.

## 1. Introduction

Energy shortages have become an urgent global issue. Against this backdrop, increasing calls for energy conservation and emission reduction have driven the rapid development of electric vehicles (EVs) [1]. With the rapid increase in EV ownership, safety issues have drawn growing attention, particularly EV fire accidents [2,3]. Statistics show that the number of EV fire accidents increased from 24 in 2016 to 276 in 2022 [4]. The main causes of EV fires include thermal runaway, electrical abuse, and mechanical abuse, such as external damage, overcharging, component short circuits, and arc faults [4,5,6].

Current research largely focuses on EV fires caused by lithium-ion battery thermal runaway or electrolyte leakage inside the vehicle [7,8,9,10]. Some studies indicate that gas particles generated after battery thermal runaway can lead to electrical breakdown and arc faults within the battery system [11,12]. The electric arc can generate high temperatures and a large amount of energy in a short period of time, which can ignite the insulation layer of the wiring and cause a fire accident. However, arc faults can also occur outside the battery system, such as on external power transmission cables [13,14]. Early studies reported that long-term mechanical compression of vehicle cables can damage insulation layers, leading to arc faults and vehicle fires [15]. EV fire accidents during charging have also been frequently reported. Zhang et al. [16] studied an improved target detection algorithm to detect fire accidents of electric vehicles at charging stations. During charging, cables experience long-term vibration and aging, increasing the risk of insulation degradation and arc faults. In DC systems, arcs lack natural zero-crossing points, making them more persistent and more likely to ignite cables and surrounding combustibles, eventually causing fires. Arc faults serve as early warning signals before EV fires. Detecting arc faults at an early stage is therefore of great importance for EV fire prevention.

Many previous studies have focused on AC arc faults in low-voltage distribution circuits. Artale et al. [17] used the chirp zeta transform to analyze high-resolution low-frequency harmonic current signals and combined appropriate indicators to identify arc faults under different load conditions. Vu et al. [18] sought multiple arc fault characteristics and combined these features through machine learning for fault classification. For DC series arc faults, the arc introduces a time-varying nonlinear impedance into the circuit, causing only a slight current drop, which makes traditional fuses and protection devices ineffective. PARK et al. [19] studied the DC arc faults in photovoltaic systems. By combining frequency domain and time domain analysis and using the impedance model, they determined the characteristic frequency bands of the arc faults to avoid interference caused by the inverter. Extensive research has been conducted on DC arc faults in photovoltaic (PV) systems, including frequency- and time-domain feature extraction and diagnostic algorithms tested on dedicated hardware platforms [20,21,22]. Arc faults may also cause fire incidents on DC micro-networks. Miao et al. [23] believe that the existing arc fault models cannot potentially represent the complex characteristics of arcs. By combining the construction of multi-feature simulation models and self-developed detection hardware, they achieved a high accuracy rate and avoided the influence of power electronic system noise. With the advancement of deep learning, multilayer neural networks have been increasingly applied to arc fault detection. Several deep neural network–based AC arc fault detection methods combined with attention mechanisms have achieved high recognition rates, such as ArcNet [24], Arc-1DCNN [25], MC-MGCNN [26], etc. Yan et al. [27] designed a band-pass filter by analyzing the frequency bands of some background noises in photovoltaic systems to avoid their interference and used a time convolutional network and a fully connected layer after PCA dimensionality reduction for the diagnosis of DC arc faults in photovoltaic systems.

However, research on DC arc fault detection in high-voltage wiring harnesses of electric vehicles remains relatively limited, especially under DC fast-charging scenarios. In DC systems, arcs lack natural zero-crossing points, making sustained arcs more likely to ignite cables and ultimately cause vehicle fires. Arc faults serve as an early warning prior to electric vehicle fires. Detecting arc faults in circuits at an early stage can effectively prevent arc-induced electric vehicle fire accidents, which is of great significance for vehicle fire prevention.

Therefore, to address these challenges, this paper aims to develop a robust and real-time DC series arc fault detection method for electric vehicle charging systems under complex noise conditions.

The main contributions of this work are summarized as follows:

(1) An improved composite arc fault model combining Cassie and Mayr models is established for preliminary analysis of arc-induced signal characteristics in EV charging systems.

(2) A real-vehicle DC charging experimental platform is constructed to collect arc fault and normal operation data under various current and SOC conditions.

(3) A novel dual-channel deep neural network architecture, Arc_TCNsformer, integrating TCN and sparse Transformer mechanisms, is proposed to achieve end-to-end arc fault detection from raw current signals.

(4) The proposed model demonstrates strong robustness against relay-induced noise and achieves real-time performance when deployed on embedded edge computing devices.

The remainder of this paper is organized as follows: Section 2 introduces a charging system arc fault model to facilitate preliminary investigation of electrical signal characteristics in the high-voltage loop between the electric vehicle power battery and the DC charger. Subsequently, a real electric vehicle is used for further experiments to better reflect practical conditions. Section 3 presents the experimental platform and current signal analysis. Section 4 introduces the deep neural network-based arc fault detection algorithm. Section 5 describes the experimental results, misclassification analysis, and deployment of the neural network on edge computing devices, and conclusions are provided in Section 6.

## 2. Arc Fault Model of the Electric Vehicle Charging System

**Figure 1:**
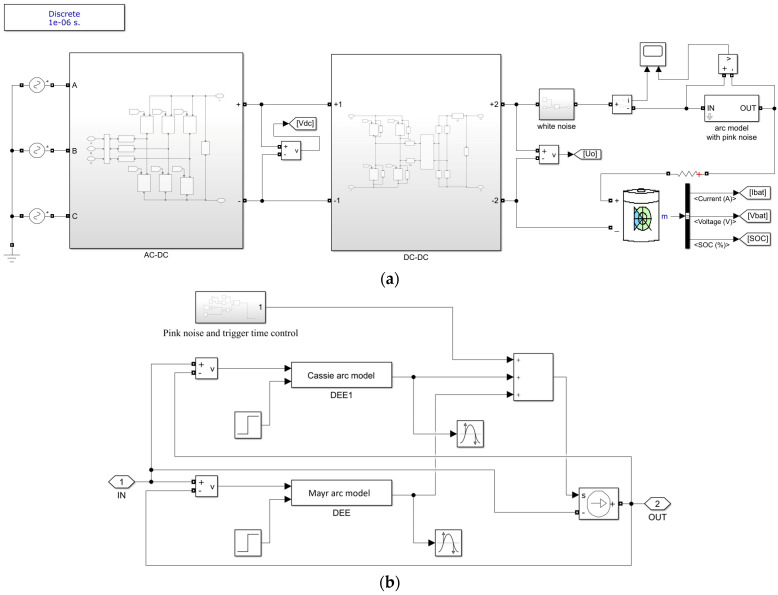
Arc Fault Model of the Electric Vehicle Charging System. (**a**) The overall topological structure of the vehicle’s charging circuit when an arc fault occurs. (**b**) A composite model combining the Cassie and Mayr models.

A charging system model includes the power grid and the DC charger. The charger circuit consists of an AC–DC conversion circuit and a DC–DC conversion circuit, as shown in Figure 1. Currently, electric vehicle chargers are mainly composed of three-phase bridge rectifier AC–DC modules and high-frequency DC–DC power conversion units. Arc faults are typical nonlinear, strongly coupled, and transient phenomena. In electrical engineering, simplified mathematical models are commonly used to describe the time-varying conductivity of arcs. The Cassie model is suitable for high-current states (steady arc burning), in which the arc voltage remains approximately constant, and the dominant mechanism is the change in conductance caused by variations in arc radius. The Mayr model is suitable for low-current states (near arc extinction), where heat dissipation dominates.

In practice, an arc experiences a transition from high current to low current and finally extinction. A single model is insufficient to describe the entire process. Therefore, a composite model combining the Cassie and Mayr models is constructed to describe the complete arc process.

(1) $$\frac{1}{g}\frac{dg}{dt}=\frac{1}{\tau_{c}}(\frac{u^{2}}{U_{C}^{2}}-1)\cdot f(i)+\frac{1}{\tau_{m}}(\frac{ui}{P_{0}}-1)\cdot (1-f(i)),$$

among them, g represents conductivity, u represents arc voltage, $U_{c}$ represents Cassie arc characteristic voltage, $\tau_{c}$ represents Cassie time constant, *i* represents arc current, $P_{0}$ represents arc column maintenance power, $\tau_{m}$ represents Mayr time constant. f(i) is the weight function.

(2) $$f(i)=\frac{i^{2}}{i^{2}+I_{0}^{2}}$$

where *i* represents arc current, $I_{0}$ is a constant. This composite arc fault model can adapt to different current levels.

The Cassie model and the Mayr model are connected in parallel to form a hybrid arc model. The input voltage passes through the arc model, and the output is current. The currents from the two models are summed and fed into the Controlled Current Source in Simulink. After passing through the Controlled Current Source, the output is converted into voltage and fed back to the input voltage, as shown in Figure 1b. By setting the time at which the arc fault is triggered in this model, the arc fault can be initiated at the specified moment during the simulation. The improved arc fault model is connected in series with the DC output of the charger supplying power to the EV battery, forming a DC series arc fault, as shown in Figure 1a.

### Simulation Analysis

**Figure 2:**
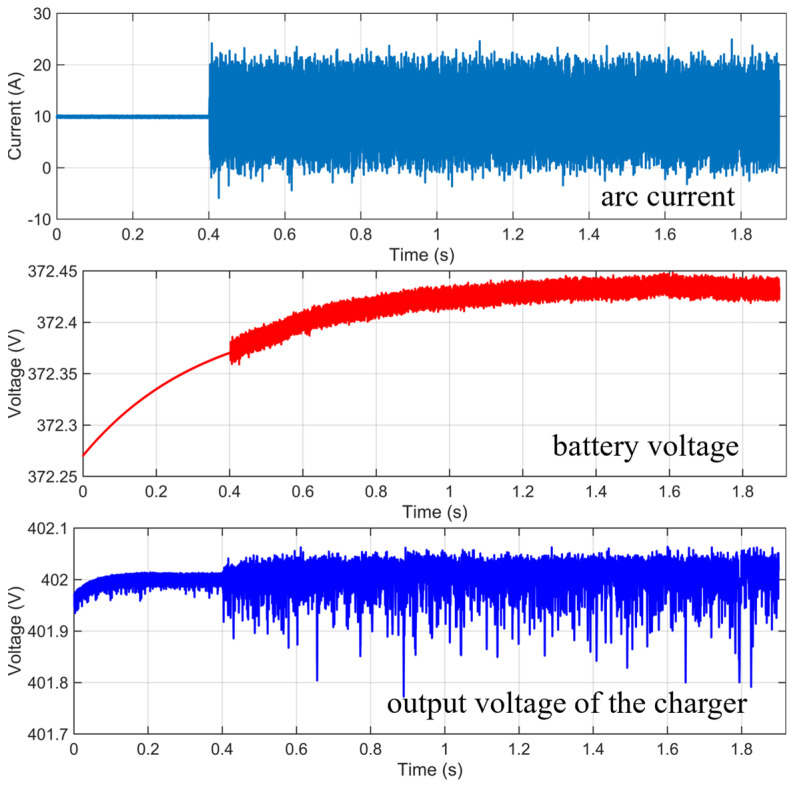
Simulation results.

With a battery SOC of 60% and a circuit current of 10 A, the simulation results are shown in Figure 2. The settings of the relevant parameters for the arc fault model are shown in Table 1. After an arc fault occurs, the current signal exhibits large nonlinear fluctuations and noise. The disturbances induced by the arc fault are also reflected in the battery voltage and the charger output voltage. Before the fault occurs, the battery voltage increases steadily and the charger output voltage shows only slight fluctuations. After the arc occurs, the high-frequency noise carried by the arc propagates into the battery, causing voltage fluctuations of a certain magnitude; meanwhile, high-frequency noise also appears in the charger output voltage, degrading its quality. If the arc persists, it is highly likely to ignite surrounding combustible materials and cause vehicle fires. Therefore, early detection of arc faults under actual vehicle charging conditions is required.

## 3. Experimental Study of DC Arc Faults During Electric Vehicle Charging

### 3.1. Experimental Platform

**Figure 3:**
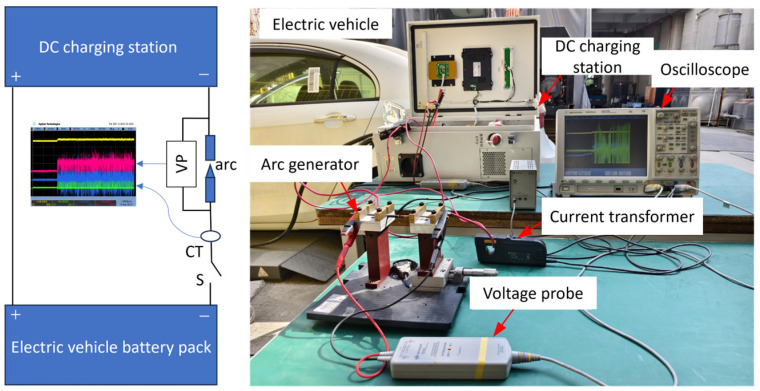
Arc fault experimental platform.

Figure 3 shows the schematic diagram and physical setup of the electric vehicle charging system arc fault experimental platform. The platform mainly consists of an electric vehicle, a 30 kW DC charger, an arc fault generation device, and a data acquisition system composed of an oscilloscope, current probes, and voltage probes. The arc generation device uses copper and carbon as electrodes. To simulate series arc faults during charging, the device complies with the UL1699B standard and adopts a point-contact design, generating arc faults by separating the electrodes. By controlling the charger output current and the battery state of charge, experiments were conducted under different currents (1–30 A) and battery SOC levels (10–90%). Data were collected for both normal charging and DC arc fault conditions. Each experiment was repeated at least three times.

### 3.2. Arc Fault Experiments in EV Charging Systems

**Figure 4:**
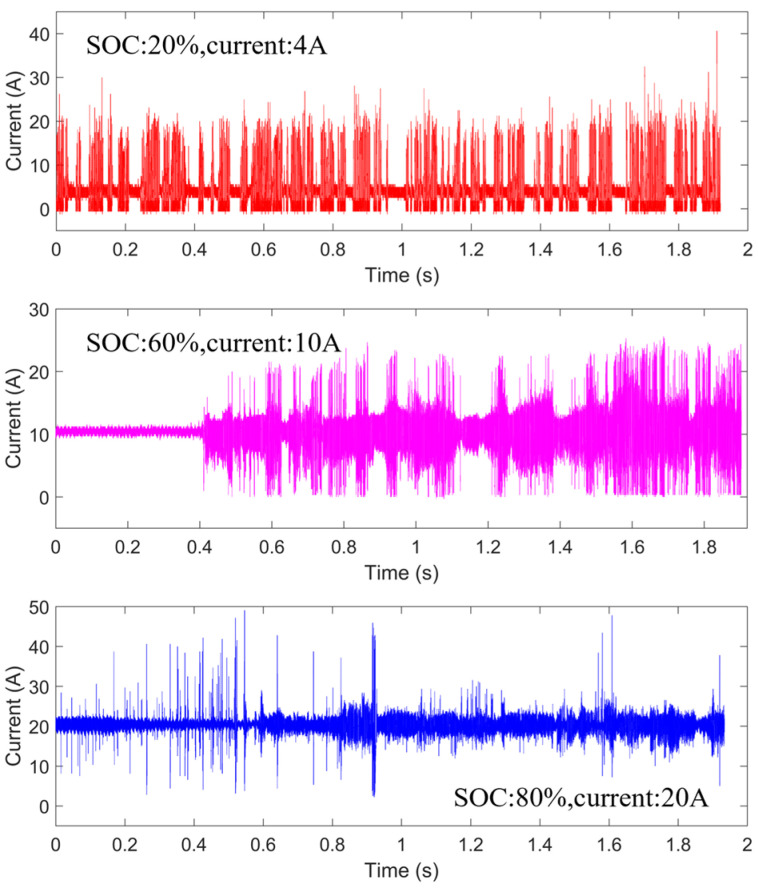
DC arc fault current signal during electric vehicle charging. During normal charging, the current waveform remains stable with minor fluctuations. After the arc fault occurs, the signal exhibits significant high-frequency oscillations and large-amplitude nonlinear disturbances, reflecting the unstable discharge behavior of the DC arc.

Figure 4 shows the experimental results of DC arc faults during electric vehicle charging. During normal operation, the current remains stable. When an arc fault occurs, the waveform exhibits severe high-frequency oscillations, reflecting the unstable current characteristics during arc discharge. The experimental results are generally consistent with the simulation results: at higher currents, the arc discharge process is more stable, whereas at lower currents the arc is unstable, repeatedly extinguishing and reigniting, resulting in sparser high-frequency noise. After an arc occurs, the charger continues charging the vehicle without any alarm indication. In real-world scenarios, after insulation damage occurs, arc faults may lead to electrical fires without timely warnings.

**Figure 5:**
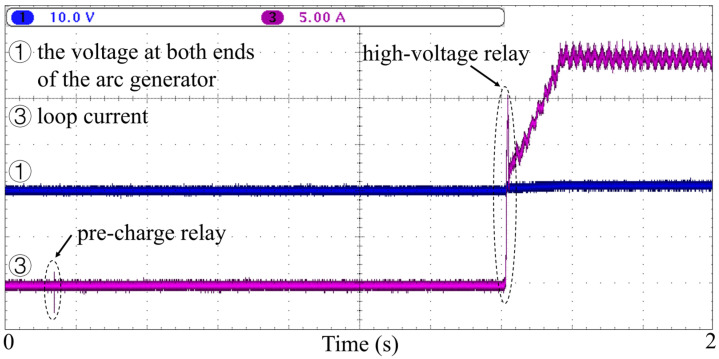
Current waveform during normal charging startup and pre-charge stage. Although no arc fault is present, high-frequency transient pulses appear due to relay switching actions in the pre-charge circuit. These noise characteristics partially resemble arc fault signals and may lead to false triggering in traditional detection methods.

It is worth noting that during the pre-charge stage of electric vehicle charging, the current signal also exhibits high-frequency noise, as shown in Figure 5. This noise is mainly caused by switching arcs generated when internal relays in the vehicle are actuated. High-voltage relays control the main circuit of the DC charging path, while pre-charge relays gradually charge vehicle capacitors through a pre-charge circuit before the high-voltage relay closes. Such conditions may cause false triggering of traditional arc fault detection algorithms or devices.

### 3.3. Time–Frequency Analysis of Charging Circuit Current Signals

**Figure 6:**
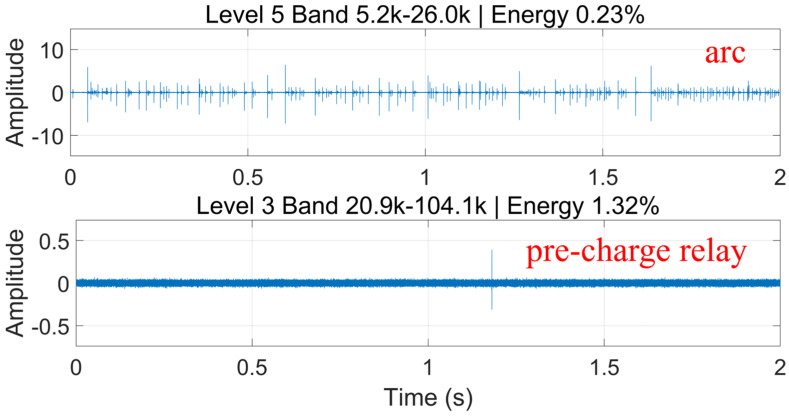
MODWT analysis of pre-charge relay and arc signals.

**Figure 7:**
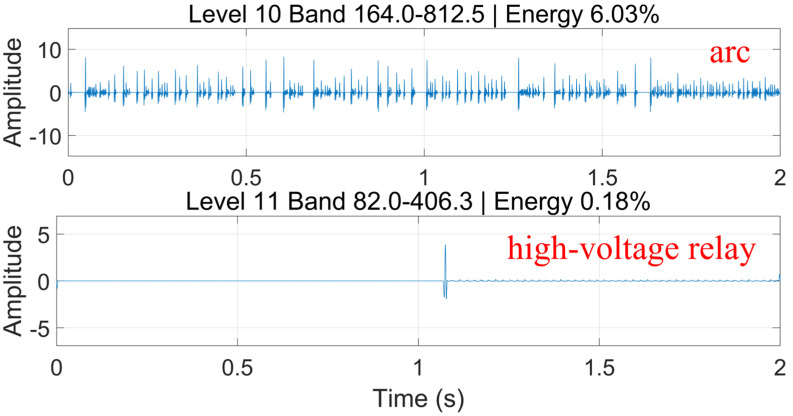
MODWT analysis of high-voltage relay and arc signals.

Therefore, time–frequency analysis was further performed on arc fault signals and relay actuation signals during normal charging. The maximal overlap discrete wavelet transform (MODWT) preserves all time-point information by eliminating downsampling, thereby enhancing translation invariance and frequency resolution. It performs well in analyzing nonstationary signals such as DC arc faults. Twelve-level MODWT analysis was conducted for both signal types. The results show that the characteristic frequency band of the pre-charge relay lies between 20.9 kHz and 104.1 kHz, while that of the high-voltage relay lies between 82.0 Hz and 406.3 Hz. These bands overlap with the characteristic frequency band of arc fault signals, as shown in Figure 6 and Figure 7. This further demonstrates the partial similarity between normal charging noise and arc fault noise. Due to the randomness of arcs and interference from charging system noise, arc fault detection methods used in other scenarios may be insufficient for electric vehicle charging systems. Deep learning has become the mainstream approach for arc fault detection, as multilayer neural networks can adaptively extract fault features and avoid reduced generalization caused by fixed feature thresholds.

## 4. Arc Fault Detection Algorithm Based on Deep Neural Networks

### 4.1. Automated Sample Labeling

**Figure 8:**
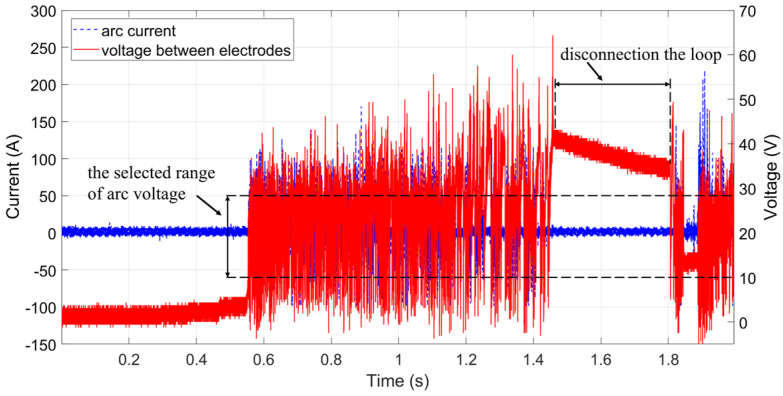
Arc voltage selection range.

Arc voltage cannot be directly used as a feature for detecting DC arc faults because arcs are random and may occur anywhere along the circuit where insulation damage or loose connections exist. Current, however, can be measured anywhere in the circuit and serves as an indicator of arc occurrence. When an arc occurs, a time-varying arc voltage is generated across the electrodes, which can be used to automatically label arc current samples. Due to arc instability, the arc may extinguish during electrode separation. At this time, the difference between the charger output voltage and the battery open-circuit voltage appears across the electrodes, as shown in Figure 8. Although a voltage is measured, it is not arc voltage. During arc events, both arc voltage and arc current exhibit significant high-frequency noise. A slight voltage increase is also observed between 0.4 s and 0.56 s due to contact resistance between electrodes. Therefore, the selected arc voltage range (indicated by dashed lines in Figure 8) avoids these regions to facilitate automated labeling of arc current.

### 4.2. Network Architecture

**Figure 9:**
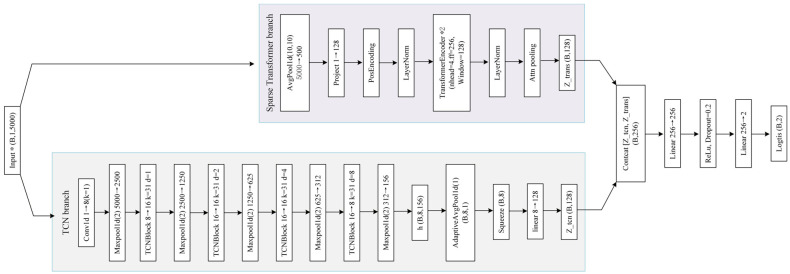
Arc_TCNsformer deep neural network architecture.

This section describes the proposed dual-channel deep network architecture, Arc_TCNsformer, shown in Figure 9. The architecture integrates a temporal convolutional network (TCN) with a downsampled sparse Transformer. The TCN captures discriminative short-term burst pulses at multiple scales, while the sparse Transformer aggregates contextual information over longer time ranges, improving robustness under complex noise conditions. The input is a single-channel current sequence of length 5000, which can be represented as $x=\{x_{t}{\}}_{t=1}^{5000}$, $x_{t}\in R$, and the output is a binary classification result, where y ∈ {0, 1}. Here, 0 represents the normal charging state, while 1 indicates the fault state of the direct current series arc.

#### 4.2.1. TCN Channel

**Figure 10:**
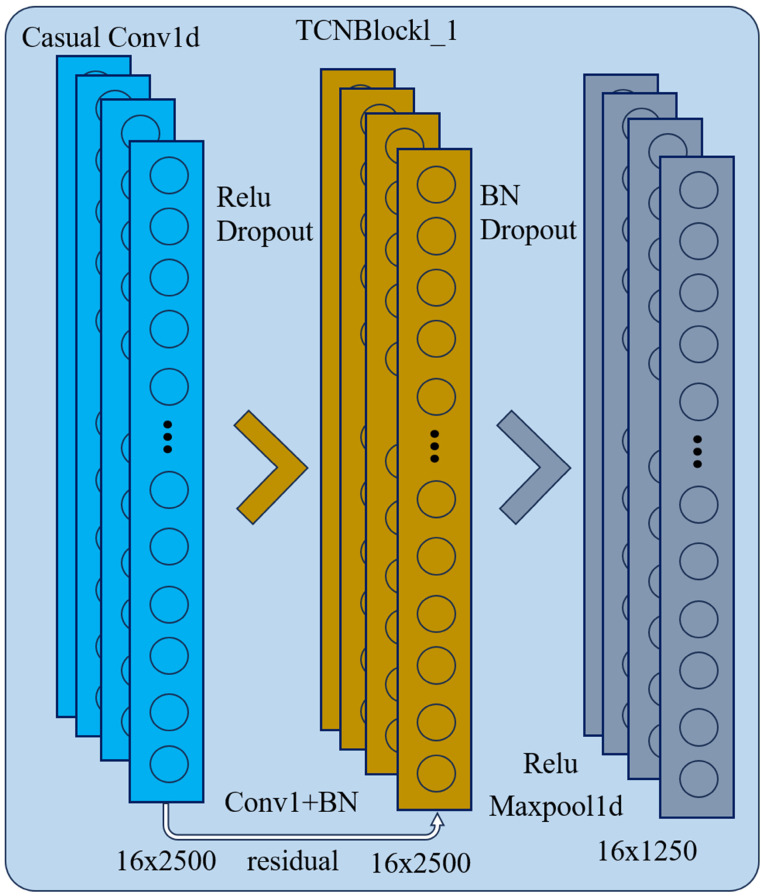
Internal structure of the TCN.

The TCN channel extracts local temporal structural features most sensitive to arc faults. To satisfy causality, causal dilated convolutions are used so that outputs at any time depend only on current and past inputs. The output of the l-th layer at time t is defined as follows:

(3) $$y_{t}^{\left(l\right)}=\sum_{k=0}^{k-1}w_{k}^{\left(l\right)}x_{t-kd_{l}}^{\left(l\right)},$$

where *k* represents the length of the convolution kernel, $d_{l}$ is the expansion coefficient, $w_{k}^{\left(l\right)}$ represents the corresponding convolution kernel weights, the initial single-channel input sequence: $x=\{x_{t}{\}}_{t=1}^{5000}$. By progressively increasing dilation factors, the convolution kernel covers a larger temporal range without significantly increasing parameters, which is effective for capturing irregular strong pulses in arc faults. As shown in Figure 10, each TCN residual block consists of two causal dilated convolution layers, batch normalization, nonlinear activation, and skip connections. Its output form is

(4) $$z^{\left(l\right)}=ReLU(BN(Conv(x^{\left(l\right)})+Skip(x^{\left(l\right)})),$$

After multiple residual blocks and pooling operations, the TCN channel outputs the feature map h with a dimension of h ∈ $R^{8\times 156}$; subsequently, adaptive average pooling is applied to compress the time dimension to 1, resulting in the channel vector v ∈ $R^{8}$, and it is linearly mapped to a 128-dimensional feature representation

(5) $$Z_{tcn}=W_{map}v\in R^{128},$$

From the perspective of receptive field, if each residual block contains two layers of convolution with a convolution kernel length of K and the expansion coefficient sequence being $\left\{d_{1},d_{2},d_{3},d_{4}\right\}$, then the effective time span of the l-th residual block without considering pooling is approximately $(2K-2)d_{l}$. Combined with multiple 5-times pooling, the TCN channel can model the short-term strong pulses caused by arc faults and their neighborhood changes within multiple scales of time. The hyperparameters of the TCN Channel are shown in Table 2.

#### 4.2.2. Sparse Transformer Channel

Although TCN excels at local feature extraction, convolution alone cannot fully exploit long-range contextual information. Arc faults during charging are not isolated events; the evolution of current waveforms before and after faults is important for distinguishing noise from real faults. Directly applying a standard Transformer to a length-5000 sequence incurs high computational cost. Therefore, one-dimensional average pooling with kernel size and stride of 10 is applied to reduce the sequence length to 500. The i-th token after downsampling is represented as

(6) $$p_{i}=\frac{1}{10}\sum_{t=10(i-1)+1}^{10i}x_{t},\;i=1,\;\ldots ,\;500,$$

After linear projection and position encoding, the input feature matrix $H\in R^{500\times d_{model}}$ is obtained. To further suppress irrelevant noise and reduce computational complexity, this paper introduces a band-limited sparse attention mechanism, which calculates the attention weights only within the time neighborhood |i−j|≤w, and suppresses the remaining positions through masking. The attention calculation form with masking is as follows:

(7) $$H^{\prime }=softmax\left(\frac{QK^{T}}{\sqrt{d_{k}}}+M\right)V,$$

where in the self-attention mechanism, the query, key, and value are $Q=HW_{Q},\;K=HW_{K},V=HW_{V}$, respectively; M is a band-shaped sparse mask matrix. The width of the attention window *w* is set to 128, and the remaining positions are set to −∞.

**Figure 11:**
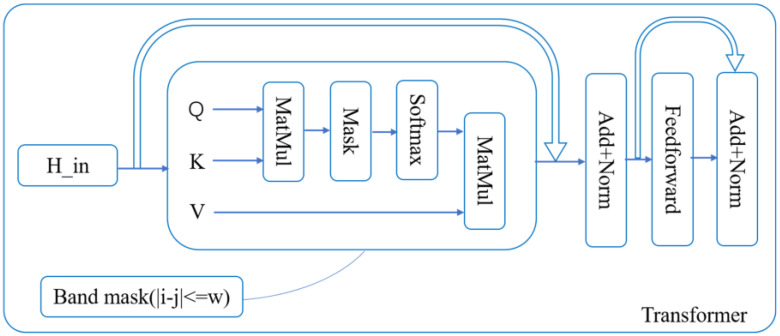
Sparse Transformer encoder.

As shown in Figure 11, this design enables the model to focus more on the neighborhood context (signals) around the time when the arc fault occurs, rather than modeling irrelevant positions globally. This helps to improve the robustness against non-stationary noise backgrounds. After two layers of encoders and layer normalization, the attention pooling method is used to aggregate along the time dimension, introducing a learnable vector a, calculating the weights of each token and performing weighted summation to obtain the Transformer channel output

(8) $$\alpha_{i}=softmax(H_{i}^{\prime }a),\;Z_{trans}=\sum_{i=1}^{500}\alpha_{i}H_{i}^{\prime },$$

where $Z_{trans}\in R^{128}$, compared to simple average pooling, this attention pooling method can automatically emphasize the time segments that are more contributive to classification, which is particularly important for the random occurrence of key disturbances in arc faults. From the perspective of computational complexity, the complexity of the standard fully connected attention is $O(B\times h\times L^{2})$, while this model uses a band-shaped mask approximation of O(B×h×L×w)(w≪L), significantly reducing the complexity. Under the conditions of L=500, w=128, it significantly reduces the memory and time costs, while maintaining the ability to aggregate neighborhood context information, making the model more suitable for subsequent deployment on edge computing devices.

#### 4.2.3. Feature Fusion and Classification

The output of the TCN channel $Z_{tcn}$ and that of the Transformer channel $Z_{trans}$ have strong complementarity at the feature level. The former focuses on local short-term pulses and multi-scale energy features, while the latter emphasizes the context relationship within the temporal neighborhood. In this paper, the two sets of features are directly concatenated to form a fused feature vector $z=[z_{tcn},\;z_{trans}]\in R^{256}$.

During the training phase, a cross-entropy loss function with category weights is adopted to alleviate the imbalance issue between the number of normal samples and faulty samples. Its form is as follows: the weights are normalized according to the inverse proportion of the number of samples of each category.

(9) $$f_{loss}=-\sum_{c\in \{0,1\}}w_{c}\cdot 1[y=c]log\overset{\wedge }{y_{c}},\;w_{c}\propto \frac{1}{N_{c}},$$

where $w_{c}$ is inversely proportional to the number of category samples. Combined with the automated labeling method in Section 4.1, the model can directly learn the discriminative patterns from the original current signal without the need for manual feature design.

To achieve batch balance sampling, an approximately balanced distribution for each batch is constructed using WeightedRandomSampler to reduce training jitter. The learning rate strategy is Warmup, with the first $E_{w}$ epochs (linearly increasing from $1\times 10^{-5}\;to\;2\times 10^{-4}$), followed by ReduceLROnPlateau (with a factor of 0.5 and a patience of 3). AMP mixed precision is used, and it automatically switches back to FP32 when encountering non-finite loss to ensure numerical stability.

## 5. Experimental Results and Discussion

### 5.1. Algorithm Performance

**Figure 12:**
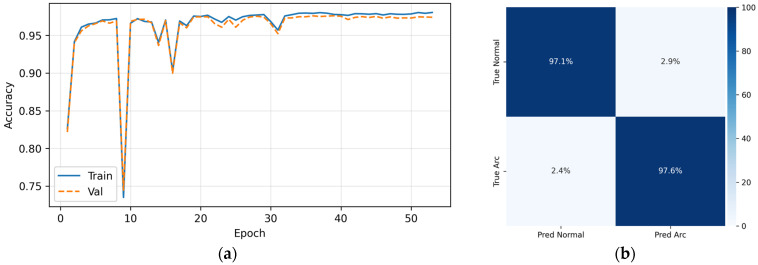
Algorithm performance comparison. (**a**) Accuracy comparison among different models, showing that the proposed TCN–Sparse Transformer model achieves the highest accuracy; (**b**) Test set classification results, demonstrating balanced recognition and strong robustness under complex charging noise conditions.

To validate the proposed model, multiple comparative experiments were conducted, as shown in Table 3 and Figure 12. Under identical datasets and training settings, Exp1 achieved the best overall performance, with an accuracy of 0.9734 and an F1 score of 0.9735. The number of false positives was significantly lower than that of other models, indicating stronger robustness under complex noise. Confusion matrix analysis shows balanced recognition of normal and fault samples, effectively suppressing misclassification caused by relay noise during pre-charge while capturing random strong disturbances in real arc faults.

### 5.2. Algorithm Deployment

**Figure 13:**
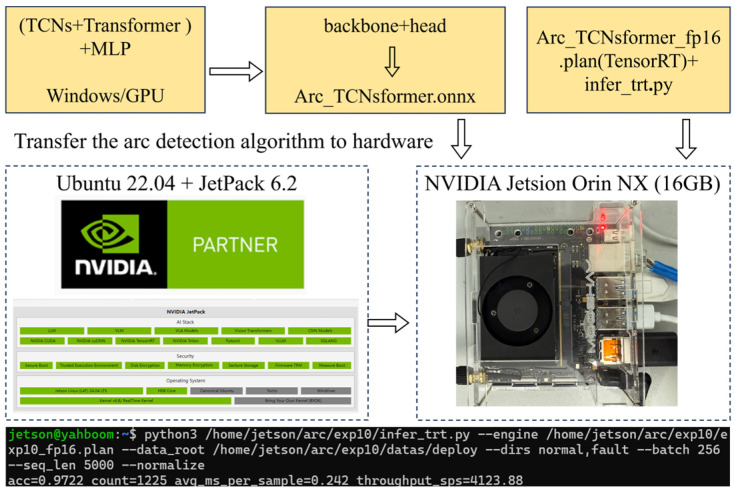
Deployment and real-time inference performance on the Jetson Orin NX SUPER platform. The optimized TensorRT FP16 engine achieves an average inference time of 0.242 ms per sample and a throughput of approximately 4123.88 samples per second.

The trained model was exported to ONNX format and deployed on a Jetson device using TensorRT v10.7 to generate an FP16 engine, as shown in Figure 13. Evaluation on Jetson Orin NX SUPER with batch size 256 yielded an accuracy of 0.9722, average inference time of 0.242 ms per sample, and throughput of approximately 4123.88 samples per second, demonstrating good accuracy and real-time performance on embedded platforms. The time performance of arc fault detection is critical because DC arcs can develop rapidly and potentially ignite surrounding materials within a short time. Therefore, the detection delay must be significantly shorter than the arc evolution time to ensure effective early warning. In the proposed method, the total detection latency consists of the signal acquisition window duration and the model inference time. For a 5000-point input window under a sampling frequency of 500 kHz, the data acquisition time is 10 ms. The deployed model achieves an average inference time of 0.242 ms per sample, which accounts for less than 2.5% of the total detection latency. This indicates that the computational delay introduced by the proposed model is negligible, and the overall detection latency remains within tens of milliseconds, satisfying practical real-time protection requirements in EV charging systems.

## 6. Conclusions

To address the difficulty of detecting DC series arc faults during electric vehicle charging and their susceptibility to confusion with normal relay-induced noise, this paper conducted arc mechanism modeling and real-vehicle experimental analysis. The conclusion is as follows:

(1) Experimental results demonstrate that the proposed Arc_TCNsformer model achieves an accuracy of 97.34% and an F1-score of 97.35%, outperforming the standalone Transformer model (94.82% accuracy) by 2.52 percentage points and the TCN-only model (96.96% accuracy) by 0.38 percentage points. Compared with the fully connected Transformer attention mechanism, the sparse attention strategy significantly reduces computational complexity while maintaining high recognition performance.
(2) Furthermore, after deployment on the Jetson Orin NX SUPER platform, the optimized TensorRT FP16 engine achieves an average inference time of 0.242 ms per sample and a throughput of approximately 4123.88 samples per second, while maintaining a deployment accuracy of 97.22%, demonstrating strong real-time capability and robustness for embedded edge applications.

These results confirm that the proposed dual-channel architecture effectively suppresses misclassification caused by relay-induced transient disturbances and provides a reliable early warning solution for electric vehicle charging safety.

## Figures and Tables

**Table 1:** Parameters of the arc fault model.

Parameters	Symbol	Numerical Value
conductivity	g	1 ×10^5^ S
arc voltage	u	Variable
Cassie arc characteristic voltage	$U_{c}$	15 V
Cassie time constant	$\tau_{c}$	1.368 × 10^−4^ s
arc current	*i*	Variable
arc column maintenance power	$P_{0}$	10 W
Mayr time constant	$\tau_{m}$	0.225 × 10^−3^ s
Constant of current	$I_{0}$	1.5 A

**Table 2:** The hyperparameters of the TCN Channel.

Hyperparameters	Numerical Value
number of residual blocks	4
kernel sizes	31
tcn1	k = 31, d = 1
tcn2	k = 31, d = 2
tcn3	k = 31, d = 4
tcn4	k = 31, d = 8

Here, k represents the kernel size, and d represents the dilation. “tcn1” represents the first TCN block.

**Table 3:** Ablation experiment of the arc fault detection model.

Exp	Exp_Model	Params	VRAM (GB)	Complexity	Accuracy	Precision	Recall	F1
1	{TCN//Sparse_Trans} + MLP (proposed model)	383 K	20–28	O(h⦁L⦁w + L⦁d_model⦁dim_ff) + O(TCN)	0.9734	0.9711	0.9760	0.9735
2	TCN + Transformer + MLP	350 K	8–12	O(h⦁$L^{2}$ + L⦁d_model⦁dim_ff) + O(TCN)	0.9714	0.9627	0.9810	0.9717
3	TCN + MLP	85 K	4–6	O(∑layers C_out⦁C_in⦁K⦁L_layer)	0.9696	0.9638	0.9760	0.9698
4	Transformer + MLP	299 K	16–24	O(h⦁$L^{2}$ + L⦁d_model⦁dim_ff)	0.9482	0.9507	0.9456	0.9482

Since all the models have MLP, the MLP part was omitted when comparing the complexities of each model. “Exp” stands for “Experiment” (abbreviation for experiment).

## Data Availability

The data that support the findings of this study are available from the corresponding author upon reasonable request. The data are not publicly available due to the nature of this research.
